# Structural Modification of Single-Layer Graphene Under Laser Irradiation Featured by Micro-Raman Spectroscopy

**DOI:** 10.1186/s11671-017-2089-6

**Published:** 2017-04-26

**Authors:** Yurii Stubrov, Andrii Nikolenko, Viktor Strelchuk, Sergii Nedilko, Vitalii Chornii

**Affiliations:** 1 V.E. Lashkaryov Institute of Semiconductor Physics National Academy of Sciences of Ukraine, 45 Nauky pr., 03028 Kyiv, Ukraine; 2 Department of Physics, Kyiv National Taras Shevchenko University, 64 Volodymyrs’ka str., 01601 Kyiv, Ukraine

**Keywords:** Graphene, Micro-Raman spectroscopy, Double electron-phonon resonance mechanism, Structural defects, Laser irradiation, Exposure dose

## Abstract

Confocal micro-Raman spectroscopy is used as a sensitive tool to study the nature of laser-induced defects in single-layer graphene. Appearance and drastic intensity increase of D- and D′ modes in the Raman spectra at high power of laser irradiation is related to generation of structural defects. Time- and power-dependent evolution of Raman spectra is studied. The dependence of relative intensity of defective D- and D′ bands is analyzed to relate the certain types of structural defects. The surface density of structural defects is estimated from the intensity ratio of D- and G bands using the D-band activation model. Unusual broadening of the D band and splitting of the G band into G^−^ and G^+^ components with redistribution of their intensities is observed at high laser power and exposition. Position of the G^+^ band is discussed in relation with nonuniform doping of graphene with charge impurities. Simultaneous presence in the Raman spectra of heavily irradiated graphene of rather narrow G band and broaden D band is explained by coexistence within the Raman probe of more and less damaged graphene areas. This assumption is additionally confirmed by confocal Raman mapping of the heavily irradiated area.

## Background

Since graphite monolayer was first produced in 2004 [1], it has become one of the most promising materials with potential technological applications [2]. Unique electronic and optical properties of graphene arise from its energy band structure with linear dispersion of electronic states near the Dirac points of the Brillouin zone (BZ). Therefore, graphene is considered as zero-gap semiconductor with electrons that behave like massless Dirac fermions [3]. Ultrahigh electron mobility (~150,000 cm^2^/V s) [4], mechanical strength (up to 1 TPa) [5], chemical resistance [6], and high thermal conductivity (up to 5300 W/mK) [7, 8] of graphene sheet make it an attractive material not only for fundamental research but also for practical applications, for example, in ultra-sensitive gas sensors [9], spintronics [10, 11], and terahertz oscillators [12] and in variety of other prospects including development of silicon electronic alternatives [3].

Graphene layers always contain structural defects that restrict and sometimes prevent their practical use. Number of defects and their type is largely dependent on the technology of graphene production [13] and can significantly affect its physical properties, such as reducing the mobility of electrons in graphene layer [14–16] or increasing its chemical reactivity [17]. Therefore, studying of structural defects is extremely important and can be used for controllable modification of physical properties of graphene by artificial creation of certain types of defects [18].

Resonance Raman spectroscopy (RRS) plays a major role in investigation of carbon-based materials being quite effective, rapid, and nondestructive method [19, 20]. The double electron-phonon resonance (DR) mechanism allows for studying one- and multi-phonon processes in monoatomic carbon layer of graphene [21]. Micro-RS can give information about nature, type [13], and concentration of defects in graphene [22, 23]. For today, there are a large number of papers devoted to investigation of structural defects in graphene generated by Ar^+^ bombardment [22, 23], fluorination, hydrogenation, mild oxidation [13], and laser irradiation [24, 25]. In particular, a time-depended structural modification of graphene by laser irradiation at low power level [25] and a pulsed laser damage of graphene were studied [24].

The present paper uses confocal micro-Raman spectroscopy as a sensitive tool to study the nature of defects in single-layer graphene induced by laser irradiation at varied laser power densities.

## Methods

### Samples

**Fig. 1:**
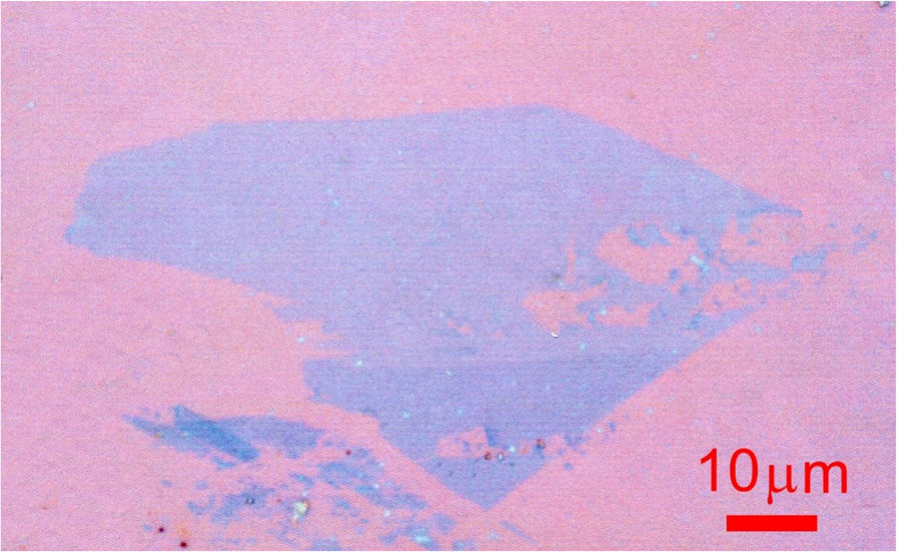
Graphene sample. Optical microscopy image of the investigated graphene sample located on Si substrate, covered with 300-nm-thick SiO_2_ layer

Studied single-layer graphene samples were prepared by mechanical exfoliation of highly oriented pyrolytic graphite. The layers were deposited onto Si substrates covered with a 300-nm-thick layer of thermal SiO_2_ for visual observation by means of optical microscopy (Fig. 1). Laser exposure of the investigated samples was performed by irradiating the samples by 488-nm laser beam with power of 0.1 ÷ 10 mW focused on sample surface by ×50/NA0.75 microscope objective, which results in power densities of ~20 ÷ 2000 kW/cm^2^.

### Raman Measurements

Micro-Raman measurements were carried out at room temperature in backscattering configuration using a triple Raman spectrometer T-64000 Horiba Jobin-Yvon, equipped with electrically cooled CCD detector. Line of Ar-Kr ion laser with wavelength of 488 nm was used for excitation. Excited radiation was focused on the sample surface with ×50 optical objectives giving a laser spot size of diameter about 1 μm. Confocal Raman mapping was performed using piezo-driven XYZ stage with a scanning step of 100 nm, ×100 optical objective, and 100-mm confocal pinhole.

## Results and Discussion

**Fig. 2:**
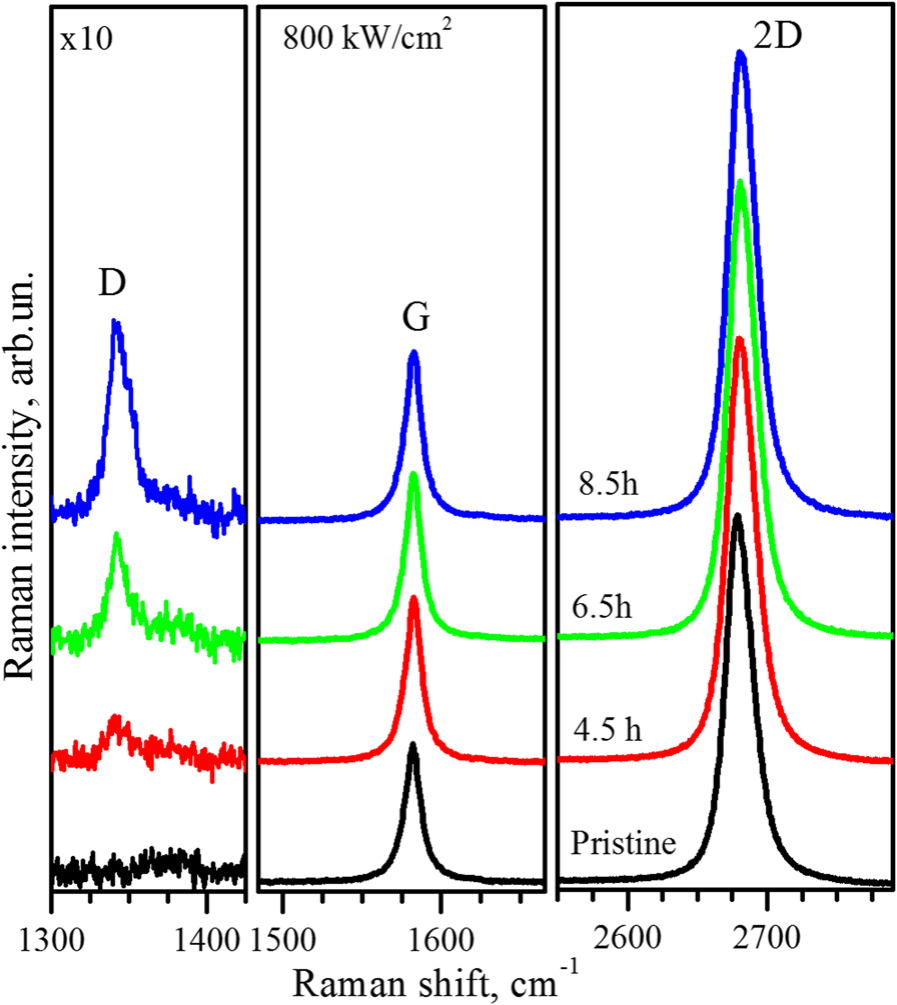
Raman spectra. Raman spectra of the pristine single-layer graphene sample and after 4.5-, 6.5-, and 8.5-h exposure by 488-nm laser with power of 800 kW/cm^2^. Spectra are normalized to the intensity of the G bands

Raman spectrum of pristine graphene sample measured at laser powers ≤800 kW/cm^2^ (Fig. 2) is typical for single-layer graphene, which is confirmed by appearance of prominent one-phonon G band at ~1600 cm^−1^, single-component two-phonon 2D band at ~2700 cm^−1^, and high *I*_2D_/*I*_G_ relative intensity [21, 26]. Appearance of these two bands in the Raman spectra are caused by scattering on Brillouin zone center phonons with *E*_2g_ symmetry and zone edge optical phonons, respectively. The physical mechanism of phonon scattering resulting in 2D band is double electron-phonon resonance (DR) process [21]. It should be noted that absence of one-phonon symmetry-forbidden D band indicates high crystal quality of the initial graphene flake.

Appearance and gradual increase in intensity of the D band at ~1350 cm^−1^ is observed in the Raman spectra of the graphene exposed to constant laser power of 800 kW/cm^2^ (Fig. 2). The origin of the D mode in graphene is well established as being defect-induced double-resonant scattering, caused by structural defect breaking of quasi-momentum conservation which allows nonzero-phonon wave vector contribution to the Raman process [27]. D band at 1350 cm^−1^ is caused by intervalley scattering process according to DR mechanism involving one of the two scattering events which occurs elastically by means of the structural defects. Thus, appearance and increase in intensity of the D band is evident in the introduction of structural defects in the graphene layer [28]. This process at relatively low laser power was registered earlier [24] and was attributed to laser-induced disassembly of the graphene layer into nanocrystalline network.

**Fig. 3:**
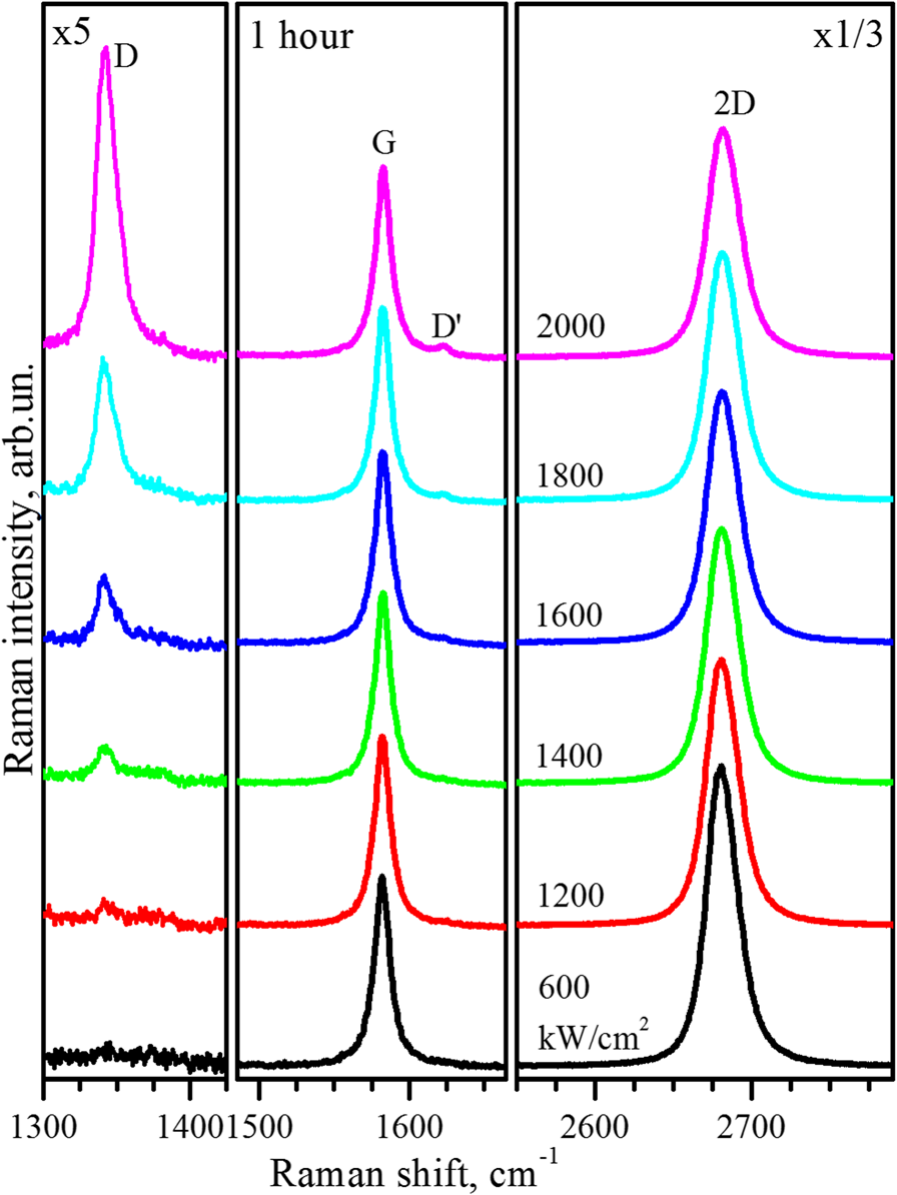
Raman spectra. Raman spectra of the single-layer graphene sequentially exposed during 1 h with varied power of 488-nm laser line. Spectra are normalized to the intensity of their G bands

Further, in order to study the power dependence of laser-induced generation of structural defects, the experiments with irradiation of the graphene with varied laser power in the range of 600 ÷ 2000 kW/cm^2^ at constant exposition time of 1 h were performed. As can be seen from Fig. 3, in addition to the D band, appearance and increase in intensity of the defect-induced D′ band at 1620 cm^−1^ is registered at laser power densities higher than 1200 kW/cm^2^. Nature of the D′ band within DR mechanism is related to intravalley scattering process, similar to D band, also involving scattering on defects [21].

**Fig. 4:**
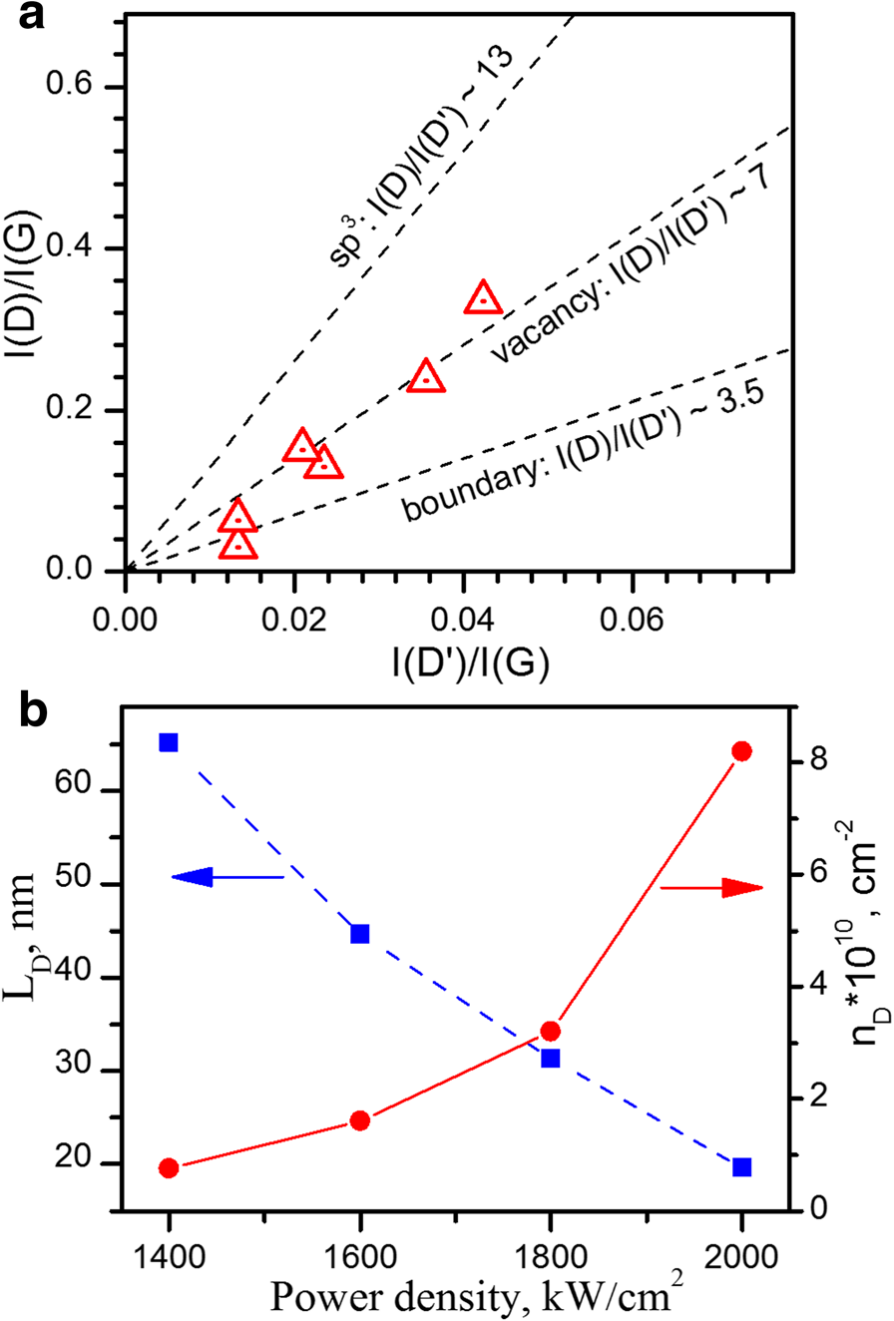
Defect characterization. **a** *I*_D_/*I*_D′_ ratio for the laser exposed graphene (Fig. 3). *Dashed lines* show *I*_D_/*I*_D′_ dependence for different type of defects [13]. **b** Estimated distance (*L*_D_) between the defects and their density (*n*_D_) on the laser power density at 1-h exposition

As it was shown in [13], type of the structural defects in graphene can be attributed from the analysis of relative intensity of D- and D′ bands. Our experimental data of *I*_D_(*I*_D′_) for laser power densities of 1400–1600 kW/cm^2^ agrees with corresponding dependence for grain-boundary defects (Fig. 4a). As the bond enthalpy for carbon-carbon single bond and double bonds makes 3.6 and 6.14 eV, respectively, two photon processes might be responsible for breaking of sp^2^ carbon-carbon bonds and alteration of the graphene lattice into nanocrystalline network [25]. At higher power densities (>1600 kW/cm^2^) the *I*_D_ to *I*_D′_ ratio have a tendency to increase toward the corresponding values found for vacancy-related and sp^3^-type defects (Fig. 4a) [13]. The energy of our laser irradiation is too low to remove carbon atom from lattice site and form a vacancy-type defects, which are known to be generated in graphene at Ar^+^ bombardment [13] or at electron beam (80–100 keV) irradiation [29]. On the other hand, we cannot rule out any chemistry on top of graphene monolayers under irradiation of high laser power such as hydrogenation and/or oxidation of graphene, which eventually changes hybridization of carbon atoms from sp^2^ toward sp^3^ and which might be the reason of increase in the *I*_D_ to *I*_D′_ ratio at high laser powers.

The average distance between structural defects can be estimated using D-band activation model [30] according to empirical relation:

1 $$ {L}_{\mathrm{D}}^2=\left(4.3\pm 1.3\right)\cdot \frac{10^3}{E_l^4}{\left(\frac{I_{\mathrm{D}}}{I_{\mathrm{G}}}\right)}^{-1} $$

where *L*_D_ is the average distance between defects (nm) and *E*_*l*_ is the energy of exciting laser radiation (eV). Using relation (1), it was found that the average distance between structural defects gradually decrease from ~65 nm down to ~20 nm when laser power density increase from 1400 up to 2000 kW/cm^2^ at constant exposition time of 1 h (Fig. 4b), which according to relation ${n}_{\mathrm{D}}={10}^{14}/\left(\pi {L}_{\mathrm{D}}^2\right)$ [30] correspond to defect density of (0.75 ÷ 8.2) ⋅ 10^10^ cm^− 2^ or relative defect concentration of (0.0001965–0.002148)%, that is sufficiently lower than those concentrations commonly used in the modeling of structural defects in graphene [31–33].

**Fig. 5:**
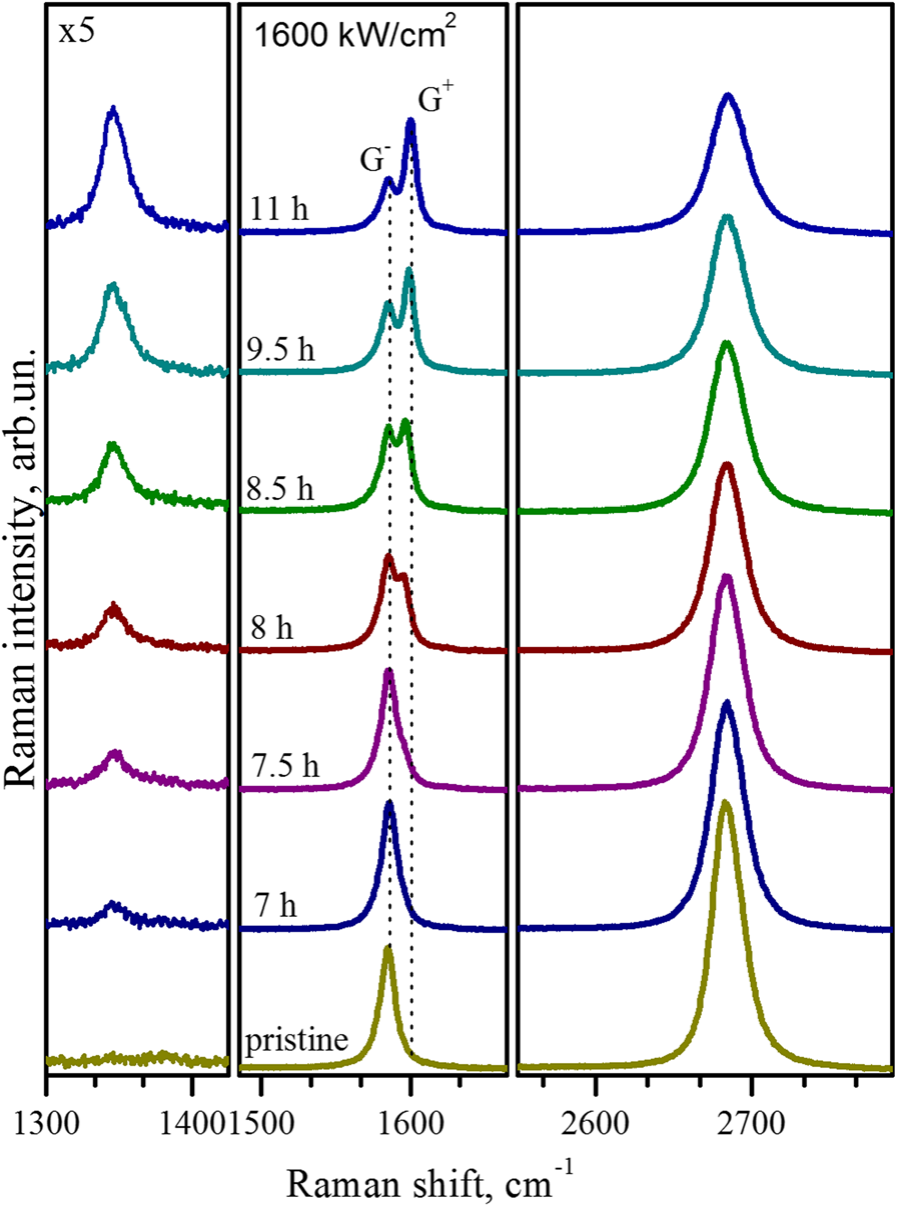
Raman spectra. Raman spectra of the single-layer graphene before and after exposure by 488-nm laser line with power of 1600 kW/cm^2^. Spectra are normalized to the intensity of the G bands

To study further the evolution of Raman characteristic bands with increase of disorder, we provided time-dependent Raman measurements of the graphene at higher power density and exposition times. The results of these measurements at power density of 1600 kW/cm^2^ are demonstrated in Fig. 5. Besides appearance and gradual increase in intensity of defect-induced D band, noticeable drop in relative intensity and broadening of 2D band and unusual splitting of the G band is also observed at higher exposures. Two prominent components peaked at ~1584 and ~1600 cm^−1^, denoted as G^−^ and G^+^ in Fig. 5, correspondingly. Relative intensity of the G^+^ to G^−^ band gradually increases with the exposition time, whereas their frequency position remains almost invariable (Fig. 5). The observed doublet behavior of Raman G band can be related to nonuniform unintentional doping of the graphene layer in the irradiation process or appearance of elastic strains. Elastic compressive strain of the graphene layer would lead to simultaneous high-frequency shift of the G and 2D bands with deformational potential of −57 and −140 cm^−1^/%, correspondingly [34], but in our case, position of the 2D band remains almost unchanged with exposition. So, elastic strains can be excluded from our consideration. Generation of structural defects in graphene due to laser irradiation can result in increasing its doping, which results in significant high-frequency shift of the G band of up to 18 cm^−1^ [35], which can be a reason for appearance of the high-frequency G^+^ component of the G band.

**Fig. 6:**
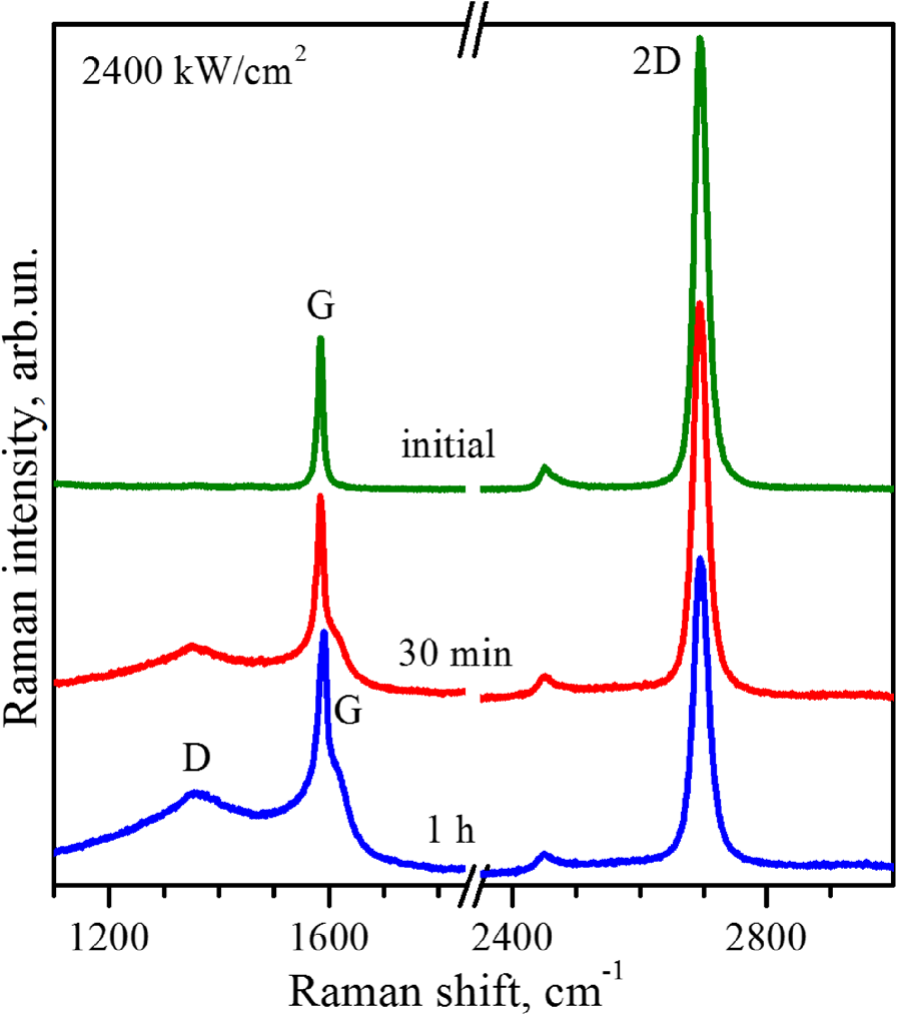
Raman spectra. Raman spectra of the single-layer graphene before and after exposure by 488-nm laser line with power of 2400 kW/cm^2^. Spectra are normalized to the intensity of the G bands

**Fig. 7:**
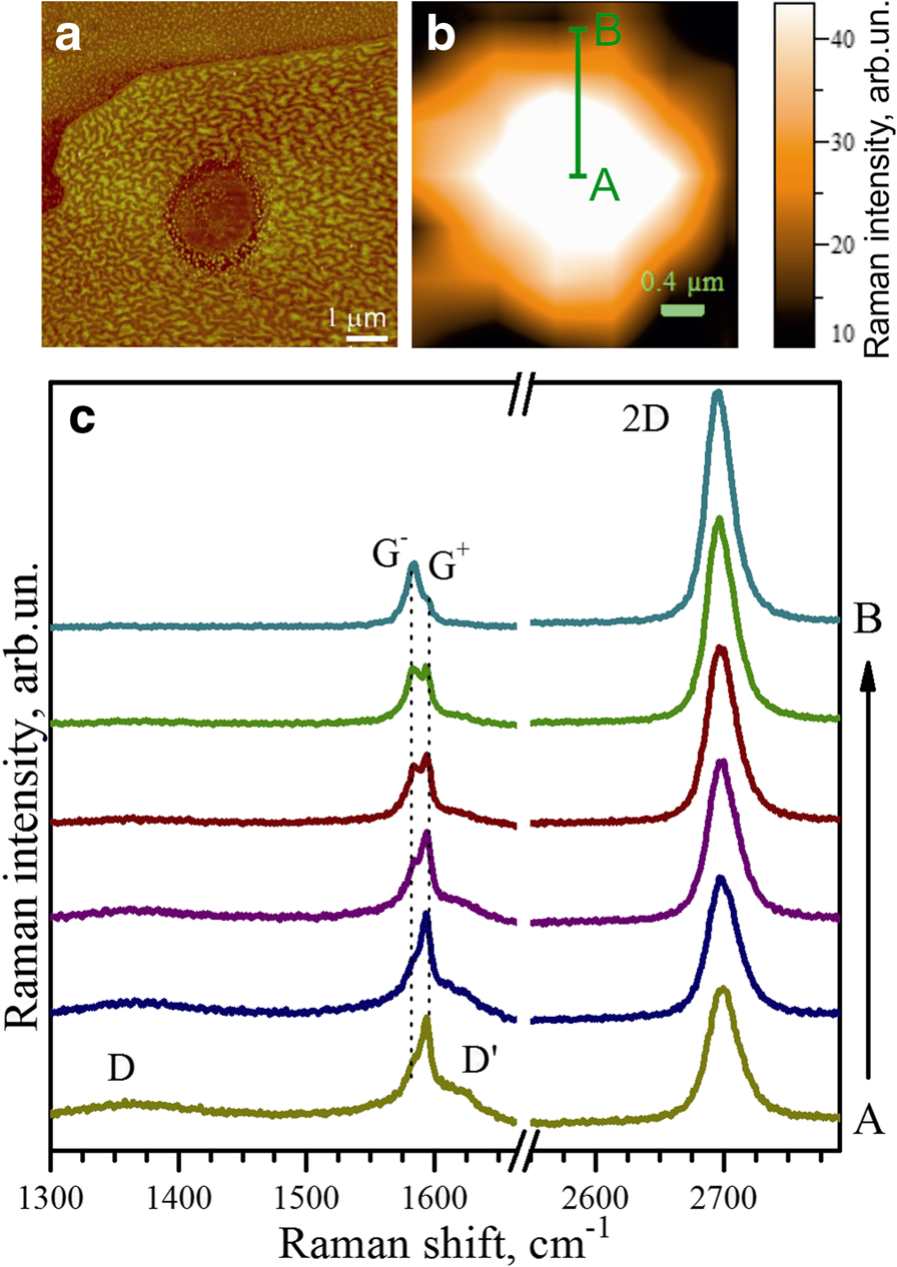
Confocal Raman mapping. **a** AFM image of the irradiated graphene area. **b** Distribution of intensity of the D band in the investigated area. **c** Set of Raman spectra obtained by scanning along the radius of the irradiated area. Irradiation was performed using 2400 kW/cm^2^ laser power and irradiation time of 1 h

Raman spectra of the single-layer graphene after laser exposure with power density of 2400 kW/cm^2^ are demonstrated in Fig. 6. Simultaneous presence in the Raman spectra from heavily irradiated area of rather narrow G band and also background consisting of broaden D- and G band components typical for amorphous carbon can be explained by coexistence within the Raman probe of more and less damaged graphene regions. To confirm this assumption, a confocal Raman mapping of the heavily irradiated area with a scanning step of 200 nm was provided (Fig. 7).

As can be seen from AFM image (Fig. 7a), the size of the damaged area is about 2 μm in diameter and there is a clear topological contrast between the irradiated and non-irradiated areas. Raman scanning from the center of irradiated area to its edge shows gradual decrease in intensity of the broadened components of the D- and G bands (Fig. 7b), increase in intensity of the G^−^ component and 2D band (Fig. 7c), and decrease in intensity of the G^+^ component of the G band. It should be noted that the spatial distribution of Raman D-band intensity in Fig. 7b is in good agreement with the AFM image of the irradiated area in Fig. 7a. These observations confirm our previous assumption about coexistence within the Raman probe of more disorder graphene in the central part of heavily irradiated area, which is featured by presence of broadened D- and G bands and also by high intensity of the G^+^ component, and less damaged area near the edge of the irradiated area, which is reflected by increase in intensity of the G band and relative intensity of the 2D band.

## Conclusions

Appearance and increase in intensity of D-like modes in the Raman spectrum of single-layer graphene at laser power ≥800 kW/cm^2^ is related to generation of structural defects. Time- and power-dependent evolution of Raman spectra is studied. The relative intensities of defective D and D′ bands for laser power densities less than 1600 kW/cm^2^ are shown to agree with corresponding dependence for grain-boundary defects and have a tendency to increase toward the corresponding values typical for vacancy-related and sp^3^-type defects at higher power densities. The surface density of structural defects is estimated from the intensity ratio of D and G bands using the D-band activation model. Unusual broadening of the D band and splitting of the G band into G^−^ and G^+^ components with redistribution of their intensities is observed during long-term laser exposure at powers densities higher than 1600 kW/cm^2^. Position of the additional G^+^ band is discussed in relation with nonuniform doping of graphene with charge impurities. Simultaneous presence in the Raman spectra of heavily irradiated graphene of rather narrow G band and broaden D band can be explained by coexistence within the Raman probe of more and less damaged graphene areas. This assumption is additionally confirmed by confocal Raman mapping of the heavily irradiated area.

## Abbreviations

BZ: Brillouin zone
DR: Double electron-phonon resonance
RRS: Resonance Raman spectroscopy
